# Pulmonary epithelial–myoepithelial carcinoma without *AKT1*, *HRAS* or *PIK3CA* mutations: a case report

**DOI:** 10.1186/s13000-020-01020-z

**Published:** 2020-08-28

**Authors:** Naoki Yanagawa, Ayaka Sato, Masao Nishiya, Masamichi Suzuki, Ryo Sugimoto, Mitsumasa Osakabe, Noriyuki Uesugi, Hajime Saito, Tamotsu Sugai

**Affiliations:** 1 Department of Molecular Diagnostic Pathology, Iwate Medical University, 2-1-1 Idaidori, Yahaba-cho, Shiwa-gun, Iwate, 0283695 Japan; 2 Department of Thoracic Surgery, Iwate Medical University, Yahaba-cho, Shiwa-gun, Iwate, 0283695 Japan

**Keywords:** Pulmonary, Epithelial–myoepithelial carcinoma, *AKT1*, *HRAS*, *PIK3CA*

## Abstract

**Background:**

Pulmonary epithelial–myoepithelial carcinoma is a rare subtype of lung cancer. Because of its rarity, the molecular information on this carcinoma is insufficient.

**Case presentation:**

We report a case of pulmonary epithelial–myoepithelial carcinoma without *AKT1*, *HRAS* or *PIK3CA* mutations in a 76-year-old woman. Computed tomography revealed a tumor located in the left lower lung. Thoracoscopic left lower lobectomy was performed. Histopathologically, the tumor consisted of duct-like structures and polygonal and spindle cell features. The duct-like structures were composed of two distinct cell layers. The inner layer consisted of cuboidal cells that were positive for pan-cytokeratin and negative for p63, whereas the outer layer consisted of polygonal and spindle cells that were positive for p63 and weakly positive for pan-cytokeratin. We evaluated mutations in *AKT1*, *BRAF*, *CTNNB1*, *HRAS*, *KRAS* and *PIK3CA* but did not detect any mutations.

**Conclusion:**

Pulmonary epithelial–myoepithelial carcinoma is a rare subtype of lung cancer, with only 56 previous cases reported in the English literature. The genetic alterations in pulmonary epithelial–myoepithelial carcinoma are still unknown. We examined the 6 genes mutation analysis, however no mutation was detected.

## Introduction

Epithelial–myoepithelial carcinoma (EMC) is a malignant tumor that occurs mainly in the salivary glands [1]. This tumor also arises in other locations such as the respiratory tract, minor salivary glands and lacrimal glands [1–3]. Primary salivary gland-type tumors of the lung account for 0.1–1% of all primary lung carcinomas, and the majority are mucoepidermoid or adenoid cystic carcinomas [4, 5]. EMC is a rare subset of salivary gland-type tumor of the lung, with only 56 cases previously reported in the English literature [6]. Because of its rarity, molecular information on this tumor type is not sufficient.

## Case presentation

**Fig. 1:**
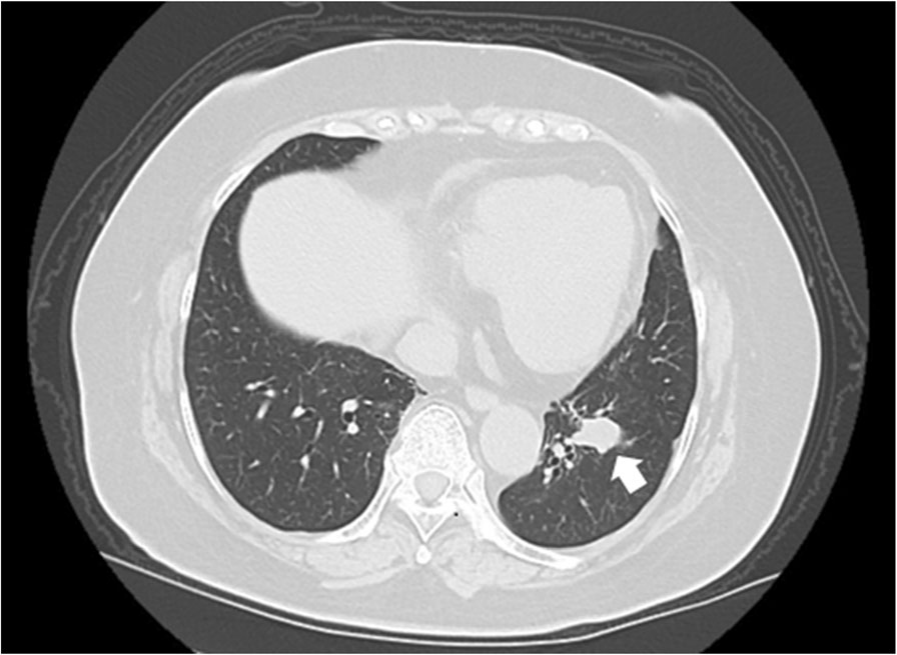
Computed tomography revealed a well-demarcated tumor, 1.8 × 1.3 cm in size, located in the left lower lung (white arrow)

A 76-year-old woman was diagnosed with colon carcinoma and underwent preoperative examinations. Computed tomography coincidently revealed a well-demarcated tumor, 1.8 × 1.3 cm in size, located in the left lower lung (Fig. 1). She did not have any respiratory symptoms. Laboratory data revealed no significantly abnormal findings. Endoscopic submucosal dissection of the colon carcinoma was performed. Bronchoscopy revealed an endobronchial mass, and transbronchial biopsy was performed. She was diagnosed with an adenocarcinoma, and thoracoscopic left lower lobectomy with hilar and mediastinal lymph node dissection was performed.

### Pathological findings

#### Macroscopic findings

**Fig. 2:**
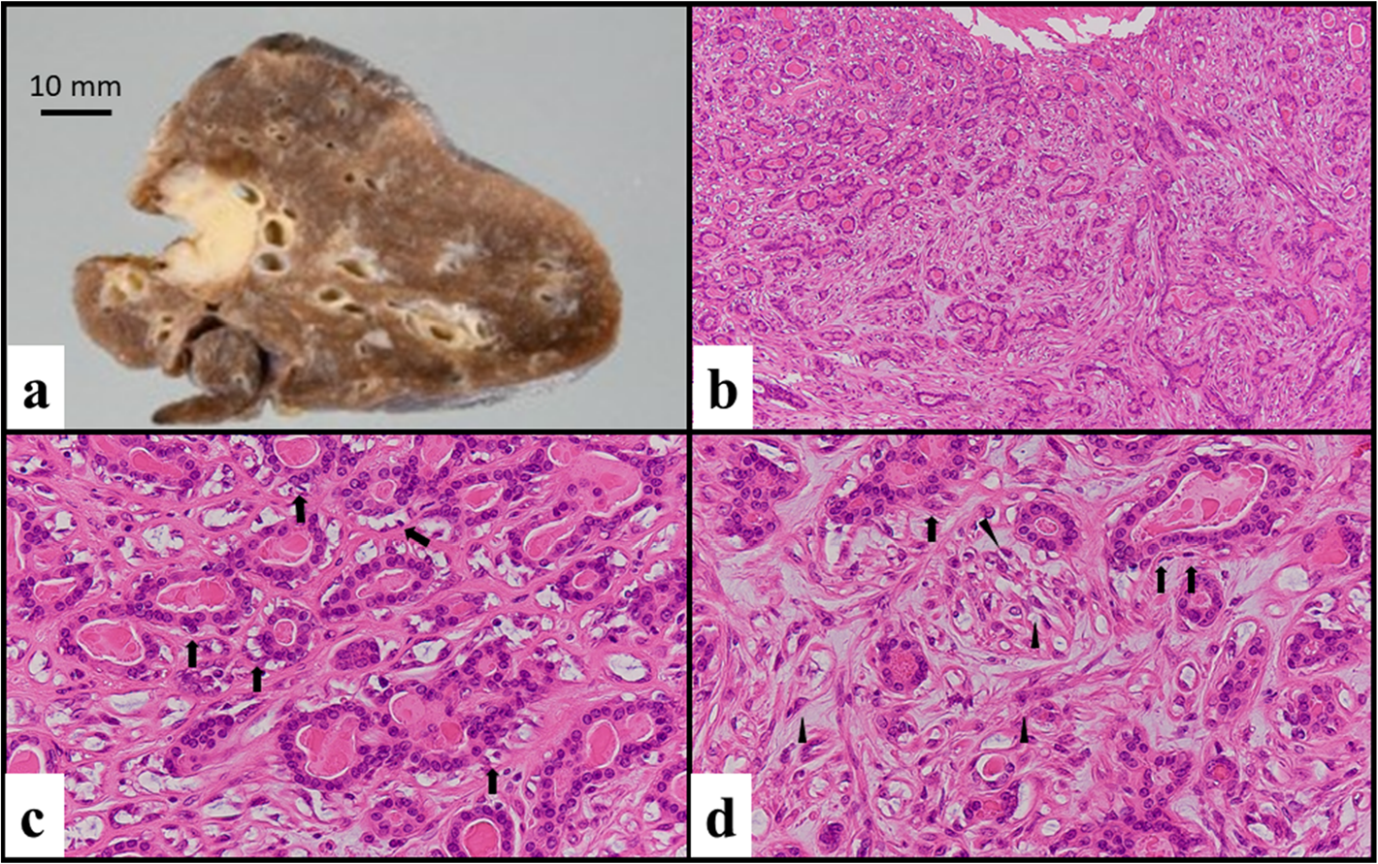
Macroscopic and microscopic findings. **a** Cut surface of the surgical specimen. The tumor size was 2.7 × 1.9 × 1.8 cm, whitish–yellow to gray, shiny and well-demarcated. **b** The tumor consisted of duct-like structures and polygonal and spindle cell features. **c** The duct-like structures comprised two distinct cell layers. The inner-layer cells were composed of cuboidal cells with eosinophilic cytoplasm and round nuclei, and the outer-layer cells (black arrow) were composed of cells with clear cytoplasm and oval-to-fusiform nuclei. **d** The polygonal and spindle cells (black arrow head) were similar to the outer-layer cells (black arrow) and had clear cytoplasm

A specimen containing the tumor was obtained at surgery. Macroscopically, the tumor size was 2.7 × 1.9 × 1.8 cm, and the cut surface of the tumor was whitish–yellow to gray, shiny, and well-demarcated (Fig. 2a).

#### Histopathological and immunohistochemical findings

**Fig. 3:**
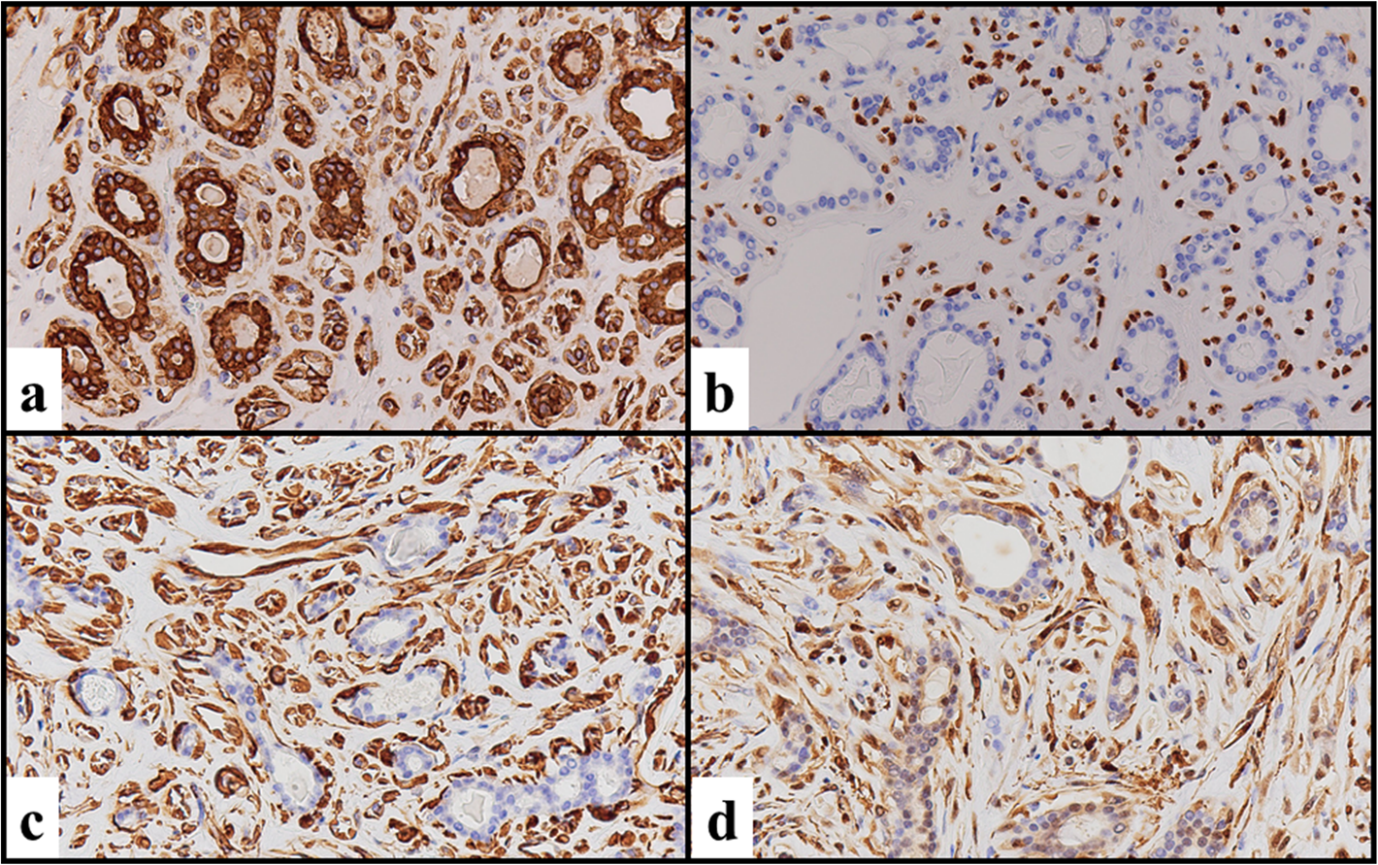
Immunohistochemical findings. **a** The cuboidal cells in the inner layer were positive for pan-cytokeratin (cytoplasm), and the outer layer of cells and polygonal and spindle cells were weakly positive for pan-cytokeratin (cytoplasm). **b**-**d** The cuboidal cells in the inner layer were negative for p63 (**b**), HHF35 (**c**) and S-100 (**d**), whereas the outer layer of cells and polygonal and spindle cells were positive for p63 (nucleus) (**b**), HHF35 (cytoplasm) (**c**) and S-100 (nucleus and cytoplasm) (**d**)

The tumor consisted of duct-like structures and polygonal and spindle cell features (Fig. 2b). The duct-like structures were composed of two distinct cell layers. The inner layer comprised cuboidal cells with eosinophilic cytoplasm and round nuclei, and the outer layer comprised cells with clear cytoplasm and oval nuclei (Fig. 2c). The duct-like structures contained eosinophilic material in the luminal spaces. The polygonal and spindle cells (black arrowhead) were similar to the outer-layer cells (black arrow) (Fig. 2d). There was no necrosis or hemorrhage. Mitotic activity was found (2 mitoses/10 high-power fields). Immunohistochemically, the inner-layer cuboidal cells were positive for pan-cytokeratin (Fig. 3a) and negative for vimentin, p63 (Fig. 3b), HHF35 (Fig. 3c), S-100 (Fig. 3d) and TTF-1, suggesting an epithelial phenotype. On the other hand, the outer-layer cells as well as polygonal and spindle cells were positive for p63 (Fig. 3b), HHF35 (Fig. 3c), and S-100 (Fig. 3d), and weakly positive for pan-cytokeratin (Fig. 3a), suggesting a myoepithelial phenotype. Overexpression of p53 protein was not found.

### Mutation analysis

We conducted polymerase chain reaction (PCR) followed by Sanger sequencing and pyrosequencing to investigate the mutation status of the oncogenes associated with EMC of the salivary gland [7]. Briefly, DNA from formalin-fixed paraffin-embedded tissues was extracted using TaKaRa DEXPAT (Takara Bio Inc., Shiga, Japan). The tumor component on the slides was microdissected to increase the tumor cell proportion. The PCR products were purified using the NucleoSpin Gel and PCR Clean-up, Mini kit (Marcherey-Nagel, Duren, Germany). Each purified product was directly sequenced using a forward primer and the BigDye Terminator version 3.1 cycle sequencing kit on the ABI 3730 instrument (Applied Biosystems Inc., Foster City, CA). Mutation analyses of *AKT1* (exon 2), *CTNNB1* (exon 3), *HRAS* (exons 2 and 3) and *PIK3CA* (exons 9 and 20) were performed based on the method described by Urano et al. [7]. The primer sequences are listed in Table 1. In addition, mutation analyses of *BRAF* (exon 15) and *KRAS* (exons 2 and 3) were performed using the BRAF Pyro Kit and KRAS Pyro Kit (Qiagen, Venlo, Netherlands), respectively, in real-time using pyrosequencing technology on the PyroMark Q24 System (Qiagen). No mutation in any of the six genes was detected.

**Table 1:** PCR primers used for Sanger sequencing

Gene	Direction	Sequence (5′ to 3′)
*AKT1* exon 2	Forward	AGGCACATCTGTCCTGGCAC
	Reverse	AAATCTGAATCCCGAGAGGCC
*CTNNB1* exon 3	Forward	TTTGATGGAGTTGGACATGG
	Reverse	AAAATCCCTGTTCCCACTCA
*HRAS* exon 2	Forward	CAGGCCCCTGAGGAGCGATG
	Reverse	TTCGTCCACAAAATGGTTCT
*HRAS* exon 3	Forward	TCCTGCAGGATTCCTACCGG
	Reverse	GGTTCACCTGTACTGGTGGGA
*PIK3CA* exon 9	Forward	TGACAAAGAAGAGCTCAAAGC
	Reverse	TTAGCACTTACCTGTGACTCCA
*PIK3CA* exon 20	Forward	TGATGACATTGCATACATTCG
	Reverse	TGTGTGGAAGATCCAATCCA

## Discussion

EMC is a rare malignant salivary gland tumor that accounts for < 1% of all salivary gland epithelial neoplasms and nearly 2% of malignant salivary gland tumors [1, 8]. EMC of the salivary gland was first described by Donath et al. in 1972 [9]. EMC is characterized by a biphasic morphology, with an inner layer of duct-like structures composed of epithelial cells and a surrounding layer of myoepithelial cells immunoreactive for S-100 and smooth muscle actin [1, 2, 6]. The tracheobronchial glands are considered counterparts of the minor salivary glands in the respiratory tract and can develop similar tumors. Within this type of neoplasia, EMC of the respiratory tract is very rare, and the diagnosis is often difficult [10, 11]. Salivary gland-type tumors of the lung account for 0.1–1% of all primary lung carcinomas, among which mucoepidermoid carcinoma is the most frequently observed histological subtype, followed by adenoid cystic carcinoma and then EMC [4, 5]. Recently, Nakashima et al. conducted a literature review of 56 patients (32 females and 24 males; average age [range], 56 [7–81] years) with pulmonary EMC [6]. Of these, 45 patients had tumors localized in the central airway within segmental bronchi appearing to be endobronchial masses. The size of the tumors varied from 0.7 to 16 cm in diameter (average 2.5 cm). According to the histopathological characteristics, three distinct histological subtypes of pulmonary EMC were reported: (1) EMC with two ductal components, defined as a characteristic feature of this tumor, (2) EMC with a solid component consisting mainly of spindle- and polygonal-shaped myoepithelial cells, and (3) EMC consisting mainly of myoepithelial cells with increased nuclear atypia, referred to as myoepithelial anaplasia [6, 12–14]. Seethala et al. reported that patients with myoepithelial anaplasia had a poorer survival compared with others [15]. Although our case was diagnosed as adenocarcinoma in the small biopsy, it proved to be typical EMC featuring two ductal components and immunohistochemically.

Several genetic mutations have been detected in EMC of the salivary glands [7, 16, 17]; *HRAS* mutations are the most frequently detected, followed by *PIK3CA* and *AKT1* mutations. The genetic alterations associated with pulmonary EMC are still unknown because of the rarity of this tumor. Urano et al. are the only ones who described HRAS mutations in all three pulmonary EMC that they analyzed [7]. However, our case did not show any mutations in *AKT1*, *HRAS* or *PIK3CA*. Although the number of reported cases is very small, the frequency of *HRAS* mutations in pulmonary EMC is 75% (3/4 cases). At this time, we cannot conclude whether the genetic alterations in pulmonary EMC are similar to those of other EMC types. Typical cases of pulmonary EMC are easy to diagnose, whereas atypical cases can be difficult to distinguish. Furthermore, because the biopsy specimen is small, the duct-like structure may be misdiagnosed as adenocarcinoma. Determination of the characteristic gene mutations will be useful for differentiating pulmonary EMC from other salivary gland tumors of the lung.

In conclusion, we report a case of pulmonary EMC containing no *AKT1*, *HRAS*, or *PIK3CA* mutations. Further examinations will be needed.

## Data Availability

The datasets used and/or analyzed during the current study are available from the corresponding author upon reasonable request.

## Abbreviations

EMC: Epithelial–myoepithelial carcinoma
PCR: Polymerase chain reaction
